# Measuring integrated hypertension care in community health centers: a cross-sectional study based on the Rainbow Model of Integrated Care in Chengdu, China

**DOI:** 10.1186/s12913-026-14845-z

**Published:** 2026-06-19

**Authors:** Yalin Zhang, Wenyan Li, Chuan Zou, Lidi Liu, Ziyu Yang, Jianchao Yang, Zhengyong Chen, Changming Liu, Can Shen, Xiaoyang Liao

**Affiliations:** 1 The Department of General Practice, West China Hospital Sichuan University Jintang Hospital, Jintang First People’s Hospital, Chengdu, Sichuan 610400 China; 2 General Practice Medical Center, West China Hospital, Sichuan University, Chengdu, Sichuan 610041 China; 3 The Department of General Practice, Affiliated Fifth People’s Hospital of Chengdu, University of Traditional Chinese Medicine, Chengdu, Sichuan 611100 China; 4 Teaching and Research Section of General Practice, General Practice Research Institute,West China Hospital, Sichuan University, Chengdu, Sichuan 610041 China; 5 School of Economics and Management, Chengdu Technological University, Chengdu, Sichuan 611730 China; 6 Department of Internal Medicine, Chengxiang Town Center Hospital Qingbaijiang District, Chengdu, 610300 China; 7 Department of General Practice, Xihanggang Community Health Center, Chengdu, 610200 China

**Keywords:** Community health centers, Integrated care, Rainbow Model of Integrated Care, Hypertension, Influencing factors

## Abstract

**Background:**

Effective hypertension management requires cross-sector coordination. The effectiveness of integrated hypertension care varies due to its multidimensional nature, yet evidence from Chinese primary care is limited. This study assessed the level of integrated hypertension care using the Rainbow Model of Integrated Care Measurement Tool (RMIC-MT) and identified key influencing factors.

**Methods:**

A cross-sectional census was conducted among all community health centers (CHCs) in Chengdu between November 2021 and January 2022. Service integration was evaluated across six dimensions: person-& community-centeredness, care integration, professional integration, organizational integration, cultural competence, and technical competence. Multilevel linear modeling was applied to identify factors associated with integration.

**Results:**

Among 110 CHCs surveyed (effective rate: 84.6%), the highest mean integration score was observed in person- & community-centeredness (4.31), whereas organizational integration received the lowest score (2.96). Higher staff education levels (β = − 0.122, 95% CI: − 0.225 to − 0.019), availability of an emergency department (β = − 0.158, 95% CI: − 0.248 to − 0.068), implementation of disease-specific outpatient management (β = − 0.170, 95% CI: − 0.288 to − 0.052), and participation in hospital alliances (β = − 0.217, 95% CI: − 0.421 to − 0.013) were positively associated with greater integration. In contrast, having ophthalmology services (β = 0.110, 95% CI: 0.009 to 0.211) and use of wearable devices (β = 0.204, 95% CI: 0.037 to 0.370) were negatively associated with integration.

**Conclusions:**

Institutional integration of hypertension services among CHCs in Chengdu remains suboptimal, with organizational domains lagging behind person-centered aspects. The findings highlight the necessity of strengthening systemic capacities—particularly in staff training, infrastructure development, and inter-organizational collaboration—to enhance the overall performance of integrated primary care.

**Supplementary Information:**

The online version contains supplementary material available at https://doi.org/10.1186/s12913-026-14845-z.

## Introduction

Hypertension affects about 33% of adults aged 30–79 worldwide and is the leading modifiable risk factor for cardiovascular diseases mortality, accounting for approximately 19% of global deaths [1]. In China, hypertension affects 27.5% of the population [2], yet blood pressure control remains strikingly suboptimal at only 5.7% [3]. This poor control drives cardiovascular and cerebrovascular diseases, which account for over 40% of annual deaths [4]. Conventional fragmented care models are ill-equipped to tackle the multidimensional complexity of hypertension; conversely, integrated care enhances control through behavioral interventions, medication monitoring, risk stratification, and coordinated referrals [5, 6].

Evidence indicates that integrated care not only lowers blood pressure and enhances health-related quality of life but also reduces hypertension-related hospitalizations and all-cause mortality [7, 8]. However, findings remain inconsistent. For instance, a multi-town clustered randomized trial in China demonstrated that an integrated care model significantly reduced blood pressure by 1.93 mmHg (95% CI: 0.063–3.8), improved self-rated health-related quality of life, and decreased hypertension-related hospitalizations by 0.17% points (95% CI: 0.094–0.24) [7]. Conversely, another study reported similar age-standardized hypertension control rates between integrated and routine care groups (50.3% vs. 52.65%, *P* = 0.518) [9]. A systematic review and meta-analysis of medical alliances in China found a significant reduction in blood pressure, though the sources of heterogeneity could not be determined [5]. Conversely, a separate meta-analysis indicated that integrated care models made little or no difference in mortality, blood pressure control, or access to care in low- and middle-income countries [10]. Such discrepancies likely reflect the absence of standardized measurement tools to capture the full scope of integrated care.

To address this, various jurisdictions have developed specific chronic disease management models emphasizing continuity and coordination, such as the Chronic Care Model(CCM), the Expanded Chronic Model(ECCM), the Innovative Care for Chronic Conditions Framework(ICCC), Rainbow Model of Integrated Care(RMIC) [11]. Among these, the Rainbow Model of Integrated Care synthesizes the theoretical foundations of integrated and primary care. Its measurement tool, developed by Valentijn through a synthesis of over 300 integration measures, is currently recognized as one of the most comprehensive assessment instruments available [12]. While recent years have seen the development of other promising tools, such as the SCIROCCO tool [13, 14], the Framework for Integrating Care [15], and the Development Model for Integrated Care (DMIC) [16], these instruments remain largely in the validation phase with limited practical application. Furthermore, a systematic review highlighted significant limitations in the current landscape of integration measurement: most tools suffer from low methodological quality, predominantly focus on narrow dimensions like person-centeredness or clinical integration, and neglect broader organizational and professional aspects. Notably, few tools capture the perspective of healthcare professionals [17].

Against this backdrop, China’s ongoing healthcare reform emphasizes integrated care through hierarchical diagnosis and treatment systems as well as medical alliance networks [18]. As a pilot city, Chengdu’s CHCs encountered significant challenges in coordinating hypertension care. To support evidence-informed policy development, this study applies the RMIC-MT to quantify the current level of hypertension care integration in Chengdu’s CHCs. We aim to: (1) quantify the current level of hypertension care integration; (2) identify modifiable institutional and general practitioner (GP)-level factors influencing integration; and (3) propose context-specific strategies to improve service delivery in line with national health priorities.

## Methods

### Study design and setting

This study was a cross-sectional census conducted among all community health centers in Chengdu, Sichuan Province, China, between November 2021 and January 2022. A multi-source survey design was adopted to evaluate the status and determinants of integrated hypertension care from both institutional and general practitioner perspectives. Chengdu, the capital of Sichuan Province in southwest China, covers an area of 14,355 km² and had a population of 21.4 million in 2023 [19, 20]. It serves as the economic and cultural hub of western China.

### Participants

A multi-stage sampling approach was used. First, all 139 CHCs in Chengdu were included. Second, GPs from each center were invited to participate. Based on the sample size estimation derived from the instrument (10 participants per item) and an anticipated 20% non-response rate, a minimum of five GPs per CHC was required. If fewer than five eligible GPs were available, all were invited. Inclusion criteria: (1) actively engaged in clinical work related to hypertension; (2) familiar with the diagnosis and treatment process of hypertensive patients; and (3) employed for at least three months. Exclusion criterion: refusal to participate. Informed consent was obtained from all participants.

### Data collection procedure

Integrated care services are shaped by multiple dimensions. A systematic review identified key determinants across patient-, professional-, and system-level domains, noting that most existing evidence—largely qualitative—comes from a limited number of review studies [21]. In this study, we primarily examined factors influencing the degree of hypertension service integration from both institutional and general practitioners (GPs) perspectives. To ensure smooth data collection, the Sichuan Provincial Health Commission distributed the questionnaires to local health authorities, who then forwarded them to the directors of community health institutions for completion.

1. Sampling and Response Rates

**Fig. 1:**
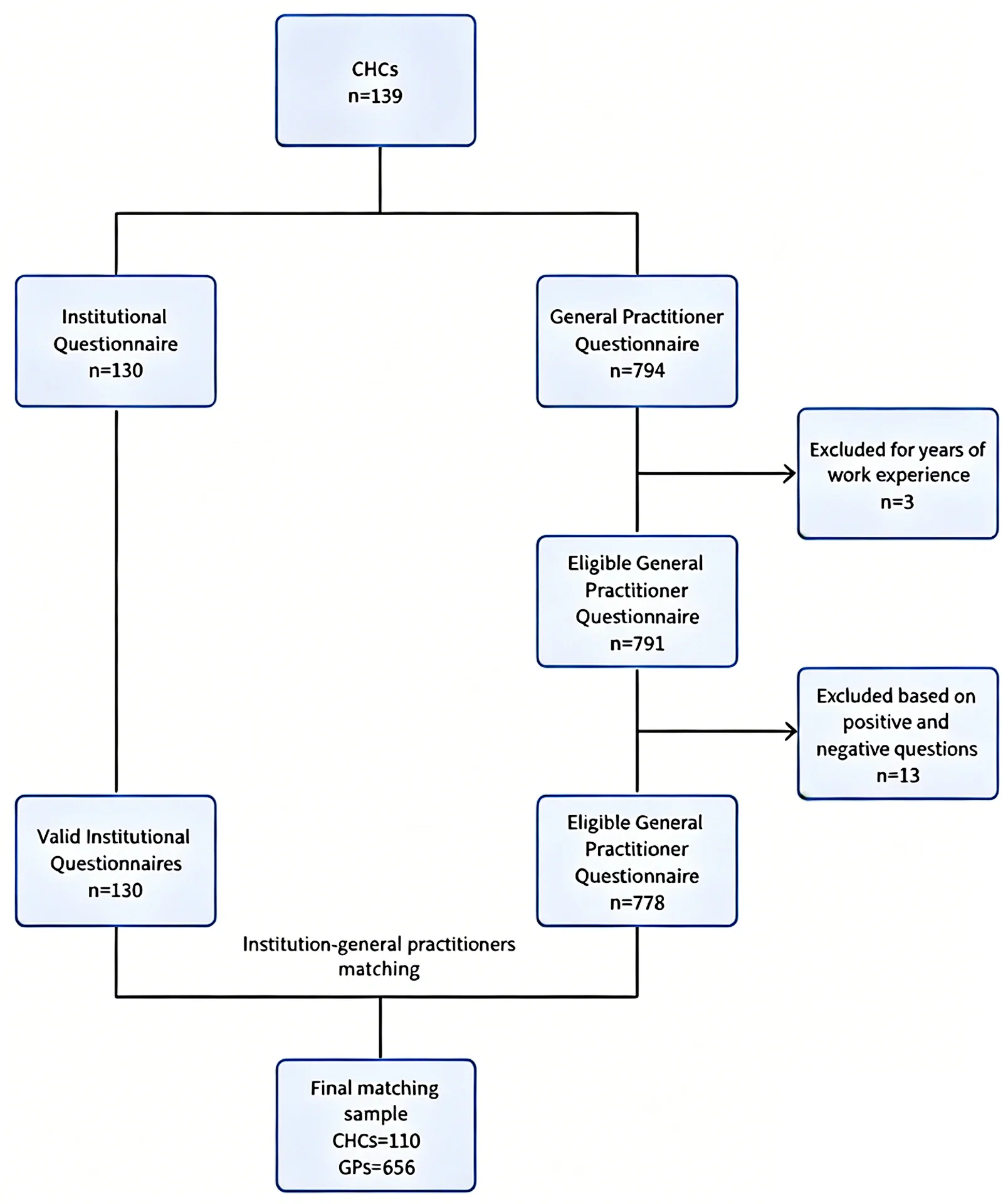
Flowchart for screening community health service centers and general practitioners in Chengdu

This survey was sent to 139 CHCs, of which 130 responded, yielding a response rate of 93.5%. A total of 794 general practitioners participated. After applying the exclusion criteria—which removed 13 questionnaires based on inconsistent responses to positive/negative questions and three due to insufficient work experience—778 questionnaires remained. After further matching these responses with their corresponding institutional surveys, the final analytic dataset comprised 656 valid General Practitioner Questionnaire (effective rate: 82.6%) and 110 Institutional Questionnaire (effective rate: 84.6%). The participant selection process is illustrated in Fig. 1.

(2) Institutional Questionnaire

A self-administered structured questionnaire was developed to evaluate the characteristics of CHCs across five domains: (1) registered service population; (2) staffing (including the number of on-duty employees, health technical personnel, their educational background, professional titles, and the number of registered general practitioners); (3) facilities and equipment (e.g., number of operational beds); (4) department setup (general practice, emergency department, and specialized departments); (5) medical services (availability of inpatient care, implementation of disease-specific outpatient management, etc.). The questionnaire was completed by institutional directors or department heads, including those responsible for clinical or administrative functions.

(3) General Practitioner Questionnaire

GPs were asked to report their experiences and perceptions of service integration in daily practice. The questionnaire was developed based on the validated Chinese version of the RMIC-MT [12]. The Chinese RMIC-MT demonstrated high internal consistency (Cronbach’s α = 0.940) and acceptable model fit (RMSEA = 0.056, SRMR = 0.061, CFI = 0.857, TLI = 0.848), confirming its reliability and validity. The instrument was used with authorization and comprised six dimensions (45 items): (1) person- & community-centeredness (9 items); (2) care integration (14 items); (3) professional integration (5 items); (4) organizational integration (4 items); (5) technical competence (5 items); and (6) cultural competence (8 items). Items in dimension 1 and 2 were rated on a 5-point Likert scale from strongly disagree (1) to strongly agree (5), while items in dimensions 4–6 were rated from never (1) to always (5). The “professional integration” domain included five reverse-coded items, scored from always (1) to never (5). Higher scores indicated stronger levels of service integration.

### Statistical analysis

Descriptive statistics were used to summarize categorical variables as frequencies and percentages, and to calculate mean scores for integration determinants and dimensions. Integration scores across RMIC-MT dimensions were visualized using radar charts. Independent-samples t-tests and one-way ANOVA were applied for univariate analyses. Multilevel linear regression was performed to identify factors associated with overall integration scores, with community health centers as level-2 units and general practitioners as level-1 predictors. Variables with *p* < 0.20 in univariate analyses were included in the final model. Variance inflation factors (VIF) < 10 indicated no multicollinearity. All analyses were conducted using SPSS version 23.0, with statistical significance set at *p* < 0.05 (two-tailed).

## Results

### Characteristics of participants

Table 1 presents the characteristics of the 656 GPs. The majority were female (69.1%), with a median age of 39 years. Regarding qualifications, 76.5% held a bachelor’s degree, and 64.3% had ten or more years of clinical experience.

**Table 1:** Demographic characteristics of general practitioners (*n* = 656)

Characteristic	*N* (%)
Gender	
Male	203(30.9)
Female	453(69.1)
Age(years)	
< 30	58(8.8)
30–49	523(79.7)
≥ 50	75(11.4)
Marital Status	
Unmarried	58(8.8)
Married	579(88.3)
Divorced	19(2.9)
Education level	
High School/Associate Degree or below	26(4.0)
Junior college	128(19.5)
Bachelor’s Degree or above	502(76.5)
Working years	
< 5	108(16.5)
5–9	124(18.9)
≥ 10	424(64.6)
Professional Title	
Junior or below	206(31.4)
Intermediate	337(51.4)
Senior Associate or above	113(17.2)

Note: Percentages may not sum to 100% due to rounding

Table 2 summarizes the characteristics of the 110 CHCs.

**Table 2:** Basic information of CHCs (*n* = 110)

Characteristic	*N* (%)
Staffing	
Percentage of healthcare technicians among total staff	
< 80%	19(17.3)
≥ 80%	91(82.7)
Percentage of licensed (assistant) with a bachelor’s degree or above	
< 50%	38(34.5)
50%-70%	36(32.7)
> 70%	36(32.7)
Percentage of healthcare technicians with an intermediate title or above	
< 35%	66(60.0)
≥ 35%	44(40.0)
Number of registered general practitioners per 10,000 people served	
< 2	67(60.9)
2–3	25(22.7)
≥ 3	18(16.4)
Facilities & Equipment	
Number of open beds	
<20	43(39.1)
20–50	28(25.5)
≥ 50	39(35.5)
CT Scanner	
No	96(87.3)
Yes	14(12.7)
Electrocardiogram (ECG) machine	
No	1(0.9)
Yes	109(99.1)
Ambulatory blood pressure monitor	
No	10(9.1)
Yes	100(90.9)
Department Setup	
General Practice Department	
No	2(1.8)
Yes	108(98.2)
Emergency Department	
No	67(60.9)
Yes	43(39.1)
Specialized Department	
No	65(59.1)
Yes	45(40.9)
Ophthalmology Department	
No	81(73.6)
Yes	29(26.4)
Medical Services	
Inpatient services provided	
No	38(34.5)
Yes	72(65.5)
Management of specific outpatient diseases	
No	21(19.1)
Yes	89(80.9)
Scheduled home visit services provided	
No	43(39.1)
Yes	67(60.9)
Established medical consortium with superior institutions	
No	6(5.5)
Yes	104(94.5)
Independent family doctor office established	
No	34(30.9)
Yes	76(69.1)
Using smartphones for disease management	
No	84(76.4)
Yes	26(23.6)
Possess wearable atrial fibrillation screening devices	
No	101(91.8)
Yes	9(8.2)

Note: Percentages may not sum to 100% due to rounding

(1) Workforce: 32.7% of CHCs had over 70% of physicians with a bachelor’s degree or higher, but only 16.4% met the national target of at least three GPs per 10,000 population.

(2) Infrastructure: 39 centers (35.5%) had 50 or more operational beds. Most CHCs (90.9%) were equipped with ambulatory blood pressure monitors, whereas only 12.7% had CT scanners.

(3) Department setup: 39.1% of CHCs had emergency departments, and 40.9% had specialized or characteristic departments.

(4) Medical services: 80.9% of CHCs provided outpatient management for specific diseases, and 94.5% participated in hospital alliances. However, only 23.6% used smartphone-based platforms for disease management.

### The level of integrated hypertension care

**Fig. 2:**
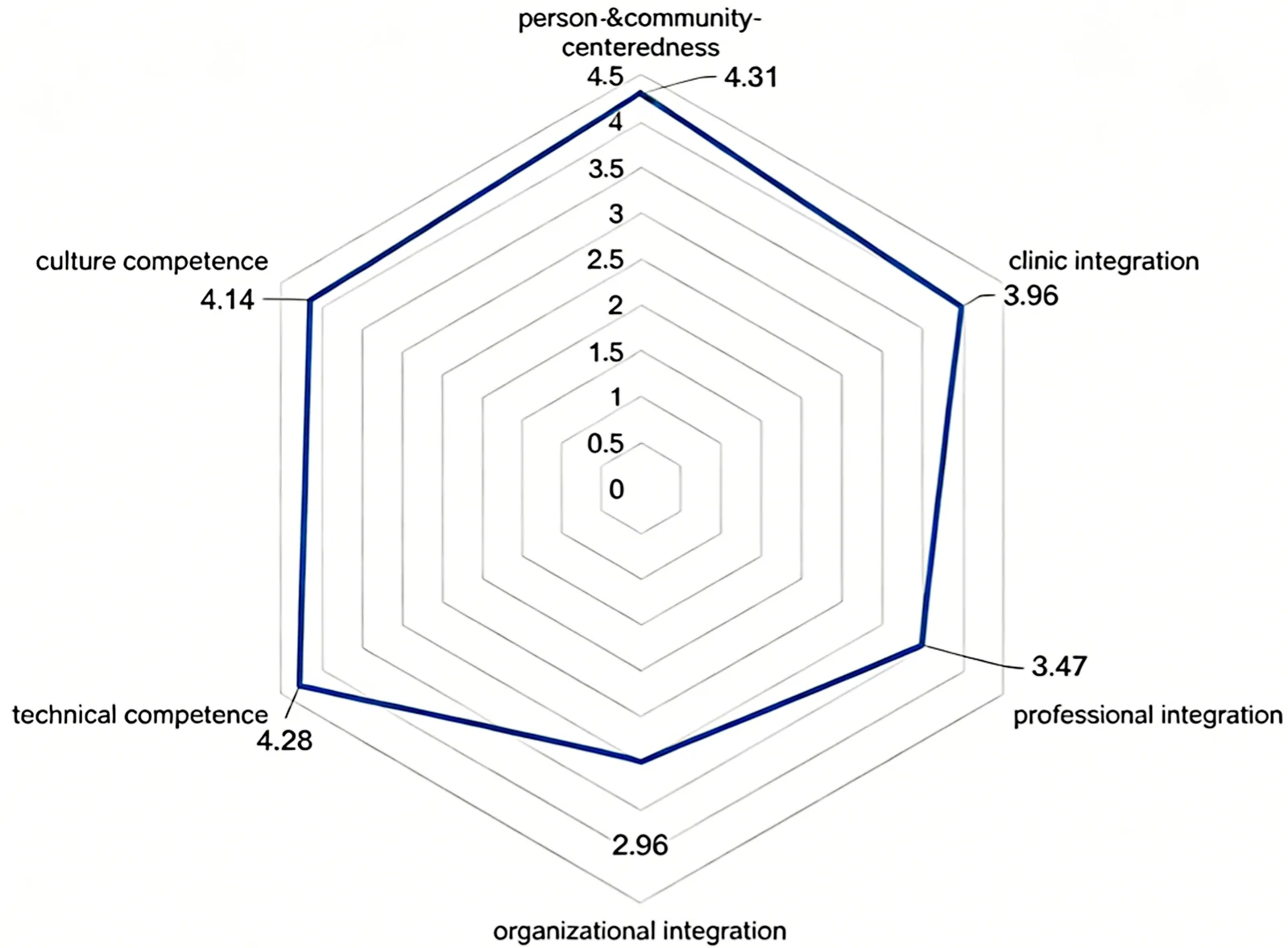
The degree of integrated hypertension care across six dimensions

The overall integration score was 3.85 out of 5. Figure 2 presents the scores for the six dimensions of integrated hypertension care, showing that person- & community-centeredness received the highest score (4.31), while organizational integration was the lowest (2.96). Among all items, “Attaching importance to building a trusting relationship with patients” scored the highest (4.61), whereas “Problems often occur during patient referrals to other medical institutions (e.g., communication failures, duplicate testing, and discontinuous treatment)” received the lowest score (2.21).

### Factors influencing integration degree

This study used the basic characteristics of CHCs and GPs as independent variables, and the overall integration score as the dependent variable, employing independent-samples t-tests and one-way ANOVA for univariate analyses. The results showed that, at the GPs level, professional title had a statistically significant effect on the degree of integration in hypertension services (*p* = 0.048). At the institutional level, four factors were significantly associated with the degree of integration: the presence of an emergency department (*p* = 0.013), implementation of disease-specific outpatient management (*p* = 0.026), participation in a medical consortium with higher-level hospitals (*p* = 0.001), and availability of wearable atrial fibrillation screening devices (*p* = 0.004) (Table 3).

**Table 3:** Single factor analysis of influencing factors of hypertension integrated service

Variable	Overall Integration Score	t/F	*p*-value
General Practitioner Level			
Gender		-1.677^a^	0.094
Male	3.80 ± 0.55		
Female	3.88 ± 0.53		
Age(years)		2.331^b^	0.098
< 30	3.99 ± 0.55		
30–49	3.85 ± 0.53		
≥ 50	3.81 ± 0.53		
Marital Status		2.078 ^b^	0.126
Unmarried	3.99 ± 0.59		
Married	3.84 ± 0.53		
Divorced	3.78 ± 0.61		
Education level		2.706 ^b^	0.068
High School/Associate Degree or below	3.92 ± 0.42		
Junior college	3.76 ± 0.54		
Bachelor’s Degree or above	3.88 ± 0.54		
Professional Title		3.048 ^b^	0.048
Junior or below	3.93 ± 0.54		
Intermediate	3.82 ± 0.53		
Senior Associate or above	3.82 ± 0.54		
** Institutional level**			
Facilities & Equipment			
Electrocardiogram (ECG) machine		1.905^a^	0.057
No	4.24 ± 0.64		
Yes	3.85 ± 0.53		
Department Setup			
General Practice Department		1.955 ^a^	0.051
No	4.14 ± 0.58		
Yes	3.85 ± 0.53		
Emergency Department		-2.481 ^a^	0.013
No	3.81 ± 0.54		
Yes	3.92 ± 0.52		
Ophthalmology Department		1.935 ^a^	0.053
No	3.88 ± 0.54		
Yes	3.80 ± 0.53		
Medical Services			
Management of specific outpatient diseases		-2.227 ^a^	0.026
No	3.76 ± 0.57		
Yes	3.88 ± 0.53		
Scheduled home visit services provided		1.380 ^a^	0.168
No	3.89 ± 0.56		
Yes	3.83 ± 0.52		
Established medical consortium with superior institutions		-3.307 ^a^	0.001
No	3.57 ± 0.64		
Yes	3.87 ± 0.52		
Independent family doctor office established		1.740 ^a^	0.082
No	3.91 ± 0.56		
Yes	3.83 ± 0.52		
Possess wearable atrial fibrillation screening devices		2.891 ^a^	0.004
No	3.87 ± 0.53		
Yes	3.64 ± 0.49		

Note: Only variables with *P* < 0.2 are given in the table; a represents the t-value of the t-test for two independent samples; b represents the F-value in one-way ANOVA

The multilevel linear model analysis demonstrated satisfactory goodness-of-fit results. The null model yielded a variance of 0.1429 at the GP level and 0.0261 at the CHC level, resulting in an intraclass correlation coefficient (ICC) of 0.1544. This indicates that approximately 15% of the variance in GPs’ evaluations of hypertension service integration was attributable to differences between CHCs. In other words, variation in integration ratings partly reflects center-level differences, underscoring the necessity of a hierarchical analytic approach (random intercept = 3.8586, *p* < 0.001). Variance inflation factors (VIFs) < 10 confirmed the absence of multicollinearity among variables. Therefore, multilevel linear modeling was appropriate for examining the factors influencing integrated hypertension services.

After sequentially incorporating independent variables from both the first (GP-level) and second (CHC-level) models—including all variables with *p* < 0.20—the − 2 log-likelihood value progressively decreased, indicating an improved model fit. The multilevel linear regression analysis identified four positive predictors of hypertension service integration: higher education level (bachelor’s degree or above vs. junior college; β = − 0.122, 95% CI: − 0.225 to − 0.019), presence of an emergency department (β = − 0.158, 95% CI: − 0.248 to − 0.068), implementation of disease-specific outpatient management (β = − 0.170, 95% CI: − 0.288 to − 0.052), and establishment of medical consortium agreements with higher-level hospitals. In contrast, the presence of an ophthalmology department and the availability of wearable atrial fibrillation screening devices (β = 0.204, 95% CI: 0.037–0.370) were identified as negative predictors of service integration (Table 4).

**Table 4:** Multilevel linear model analysis of influencing factors of integrated hypertension care1

	β	Std. Error	t	*p*	95%CI
**Fixed Effects**					
Intercept	3.694	0.086	42.976	<0.001	(3.523, 3.865)
**Level 1**					
Education Level (Ref: Bachelor’s degree or above)					
High school or technical secondary school and below	0.079	0.107	0.743	0.458	(-0.130, 0.289)
Junior college	-0.122	0.052	-2.335	0.020	(-0.225, -0.019)
**Level 2**					
Ophthalmology Department (Ref: Yes)					
No	0.110	0.050	2.180	0.033	(0.009, 0.211)
Emergency Department (Ref: Yes)					
No	-0.158	0.453	-3.488	0.001	(-0.248, -0.068)
Provision of Special Disease Management in Outpatient Clinics (Ref: Yes)					
No	-0.170	0.059	-2.863	0.005	(-0.288, -0.052)
Signed Medical Consortium Agreement with Higher-level Medical Institutions (Ref: Yes)					
No	-0.217	0.102	-2.124	0.037	(-0.421, -0.013)
Availability of Wearable Atrial Fibrillation Screening Devices (Ref: Yes)					
No	0.204	0.084	2.430	0.017	(0.037, 0.370)

## Discussion

Consistent with studies conducted in Guangxi, China [22], and in mental health services in New South Wales (NSW), Australia [23], our findings reveal a paradox: while person- & community-centeredness achieved the highest score (4.31), organizational integration remained notably low (2.96). This disparity likely reflects the structural disconnect inherent in China’s primary healthcare system. While frontline providers excel at delivering patient-centered interactions (“micro-level” integration), the organizational integration required to translate these efforts into systemic efficiency remains deficient. As defined by the RMIC framework, organizational integration serves as the critical intermediary mechanism linking macro-level policy intentions (e.g., medical alliances) to micro-level care delivery. Our results signal a failure in this bridging function, manifesting as: (1)Poor Interoperability: Lack of connectivity between medical and public health systems.(2)Limited Data Exchange: Inability to share lab/imaging results via systems or alternative channels.(3)Low Outcome Transparency: Inadequate access to post-discharge outcomes (e.g., satisfaction, mortality).(4)Restricted Accessibility: Difficulty retrieving patient data from other facilities. Without these structural linkages, high person-centeredness remains confined to individual encounters, hindering effective system-wide implementation [24, 25].

Moreover, previous studies have reported that “functional integration” scored lowest in integrated care models for pregnant and postpartum women, patients with dementia, and those with kidney disease, while the highest-scoring dimensions differed across service types [25–27]. This suggests that the extent of integration varies by service domain, and thus integration priorities should be tailored to each field. Accordingly, different integration strategies should be developed based on contextual needs and service characteristics. In Chengdu, it is essential to strengthen inter-institutional collaboration by establishing formal cooperation mechanisms, including collaboration agreements, performance distribution frameworks, and shared human and material resource plans. Integration efforts should focus on the primary care level, promoting both horizontal and vertical coordination of health resources from the community upward, in line with the health service needs of local residents [28].

This study examined the CHC-level and GP-level factors influencing the degree of integration in hypertension services. The results indicated that higher educational attainment (bachelor’s degree or above compared with junior college), establishment of emergency departments, implementation of disease-specific outpatient management, and participation in medical alliances with higher-level hospitals were four positive predictors of integration. In contrast to our findings, Chen Siyuan reported that in Guangxi’s pilot medical alliance counties, medical staff with secondary vocational education or below rated service integration higher than those with bachelor’s degrees [22]. A possible explanation is that more highly educated practitioners possess a deeper understanding of integrated care concepts, making them more capable of engaging in and critically evaluating integration practices. Furthermore, institutions with emergency departments demonstrated stronger integration performance, likely because, during the COVID-19 prevention and control period, emergency departments functioned as key hubs linking primary and tertiary hospitals, thus facilitating service coordination across care levels [29].

In addition, special disease management within outpatient departments was associated with higher integration levels, as it can reduce patients’ economic burden and support standardized, long-term management of chronic conditions [30]. Consistent with previous systematic reviews, integrated services are influenced by multiple interacting factors, including organizational, financial, and governance dimensions, as well as patient- and professional-level determinants [21]. The factors influencing integration are multifaceted and context-dependent. Future research should therefore explore these determinants across multiple system levels to better understand and optimize integrated care implementation.

The negative associations observed between wearable device use, ophthalmology departments, and integration should be interpreted with caution. Although health technologies are typically designed to enhance service integration, their fragmented or uncoordinated implementation—for instance, introducing devices without adequate staff training or workflow integration—may divert resources from essential team-based activities. This aligns with Dean F. Sittig’s sociotechnical model, which highlights the interdependence between technology and eight key dimensions, including people, workflows, and organizational context; when these elements are misaligned, negative consequences can occur [31]. Furthermore, a systematic review reported that while wearable devices produced modest improvements in certain outcomes, their effectiveness varied substantially, and many studies demonstrated poor long-term adherence [32]. These findings suggest that merely introducing devices without embedding them within a coordinated care system has limited impact, and may even waste resources if the generated data are not effectively utilized in continuous, team-based care processes.

## Limitation

This study applied the RMIC-MT to evaluate the degree of integration of hypertension services in Chengdu’s CHCs, providing a systematic and comprehensive framework for understanding integrated care performance. However, the analysis primarily focused on general practitioners’ demographic characteristics and the institutional factors of staffing, facilities, department configuration, and medical services, without including other potential determinants such as policy, financial, or patient-related factors, which limits the comprehensiveness of the findings. Moreover, the cross-sectional design restricts the ability to infer causal relationships between influencing factors and integration levels. Regarding sample representativeness, although the institutional response rate was high (93.5%), 20 of the 130 responding CHCs were excluded from the final analysis due to matching failures. While comparisons indicated no significant differences in the majority of institutional characteristics, nor in any of the key predictor variables included in our regression models, the exclusion of these centers may still limit the generalizability of our findings, particularly to specific subgroups of smaller or resource-limited facilities. Finally, as all participants were drawn solely from CHCs in Chengdu, the generalizability of the results to other regions or healthcare settings may be limited.

## Conclusion

Integrated hypertension care in Chengdu’s CHCs exhibits strong person-centered orientation but weak organizational integration. Higher educational attainment and robust institutional infrastructure emerged as key enablers of integration, such as improve the educational level of professionals, establish emergency departments, conduct management of special outpatient diseases, and strengthen the construction of medical alliances can help enhance the degree of integrated services.

## Supplementary Information

Below is the link to the electronic supplementary material.


Supplementary Material 1: Institutional Questionnaire and General Practitioner Questionnaire


## Data Availability

All the data are available from the corresponding author via email on reasonable request.

## Abbreviations

CCM: Chronic Care Model
ECCM: The Expanded Chronic Model
ICCC: The Innovative Care for Chronic Conditions Framework
DMIC: The Development Model for Integrated Care
CHCs: Community health centers
RMIC: Rainbow Model of Integrated Care
RMIC-MT: Rainbow Model of Integrated Care Measurement Tool
GPs: General practitioners
CI: Confidence Interval
ICC: Intraclass correlation coefficient
NSW: New South Wales
