# Supplementary material for: Measuring integrated hypertension care in community health centers: a cross-sectional study based on the Rainbow Model of Integrated Care in Chengdu, China
Source: BMC Health Serv Res. 2026 Jun 19;26:1224. doi: 10.1186/s12913-026-14845-z (PMC13536832; doi:10.1186/s12913-026-14845-z)
Supplement: Supplementary file 1 [file 12913_2026_14845_MOESM1_ESM.docx]

**Survey on the Status Quo of Community Health Service Centers in Chengdu**

- **Target Institutions**: All Community Health Service Centers (CHSCs) within Chengdu.
- **Purpose**: This survey is solely for scientific research purposes. The data collected will **not** be used as an administrative performance indicator. Please provide accurate and truthful responses.
- **Survey Structure**: The assessment consists of two parts:
  1. Part I: Assessment of the Basic Medical Service Capacity of CHSCs in Chengdu (**Institutional Questionnaire**).
  2. Part II: Survey of General Practitioners in CHSCs in Chengdu (**General Practitioner Questionnaire**).

**Filling Instructions**

**I. Assessment of the Basic Medical Service Capacity of CHSCs in Chengdu (Institutional Questionnaire)**

1. Reporting Period: Data should reflect the period from January 1, 2020, to December 31, 2020.
2. Unique ID Numbers:
   - Each district/county has a unique 6-digit ID (e.g., Jinjiang District: "510104", Dayi County: "510129"). The District/County ID must be 6 digits.
   - Each institution has a unique 8-digit ID (e.g., CHSC of B Road, A District: "51010401"). The Institution ID must be 8 digits. Please verify carefully before filling.

**II. Survey of General Practitioners in CHSCs in Chengdu (General Practitioner Questionnaire)**

- **Target Respondents**: At least **5** general practitioners from each CHSC should complete this questionnaire. If there are fewer than 5, all GPs should participate.
- **Eligibility Criteria**: GPs should be actively involved in hypertension-related clinical work, familiar with the clinical diagnosis and treatment process for hypertensive patients, and have worked at the center for at least 3 months.

**（1）Assessment of the Basic Medical Service Capacity of CHSCs in Chengdu**

- Institution Name: ____________________
- Institution ID (Refer to Attachment 2): ____________________
- District/County: ____________________
- District/County ID (Refer to Attachment 2): ____________________
- Designated Population Served: □□. □□ (in ten thousand)

**1. Staffing**
1.1 Total number of active staff: ___________
1.2 Number of healthcare technicians: ___________ (of which, bachelor's degree or above: ___________)
1.3 Number of licensed physicians: ___________
1.4 Number of licensed assistant physicians: ___________
1.5 Number of licensed (assistant) physicians holding a bachelor's degree or higher: ___________
1.6 Number of registered general practitioners: ___________

**2. Clinical Department Setup** (Please mark ☐)
2.1 General Practice Department: ☐ No ☐ Yes
2.2 Emergency Department: ☐ No ☐ Yes
2.3 Specialized Department: ☐ No ☐ Yes
2.4 Ophthalmology Department: ☐ No ☐ Yes

**3. Facilities and Equipment**
3.1 Number of open beds: ___________ beds
3.2 CT Scanner: ☐ No ☐ Yes
3.3 Electrocardiogram (ECG) Machine: ☐ No ☐ Yes
3.4 Ambulatory Blood Pressure Monitor: ☐ No ☐ Yes

**4. Medical Services**
4.1 Provision of inpatient services: ☐ No ☐ Yes
4.2 Management of specific outpatient diseases: ☐ No ☐ Yes
4.3 Provision of scheduled home visit services provided: ☐ No ☐ Yes
4.4 Established medical consortium with superior institutions: ☐ No ☐ Yes
4.5 Established independent family doctor office: ☐ No ☐ Yes
4.6 Use of smartphone applications for disease management: ☐ No ☐ Yes
4.7 Possession of wearable atrial fibrillation screening devices: ☐ No ☐ Yes

**(II) Survey of General Practitioners in CHSCs in Chengdu**

Dear General Practitioner Colleague:

We are conducting an assessment survey on the care coordination capacity related to hypertension management services in Community Health Service Centers. The aim is to identify areas for improvement and guide efforts to enhance medical service capabilities.

This questionnaire is for academic research purposes only. All information provided will be kept strictly confidential. Please feel assured in your responses. Thank you for your participation and support!

1. District/County of your institution: _________________________________

2. Name of your institution: _________________________________

3. Gender: ☐ Male ☐ Female

4. Age: __________ years

5. Marital Status: ☐ Unmarried ☐ Married ☐ Divorced

6. Education Level: ☐ High School/Associate Degree or below ☐ Junior college

☐ Bachelor's Degree or above

7. Years worked in current position: ____________ years

8. Professional Title: ☐ Junior or below ☐ Intermediate ☐ Senior Associate or above

9. Personal Emphasis (Please mark the appropriate box for each row)

| Statement | Strongly Disagree | Disagree | Neutral / Don't Know | Agree | Strongly Agree |
| --- | --- | --- | --- | --- | --- |
| Establishing trusting relationships with patients |  |  |  |  |  |
| Initiating communication with patients to understand their health needs |  |  |  |  |  |
| Understanding the patient's family, work, hobbies, and social situation |  |  |  |  |  |
| Understanding the patient's expectations regarding treatment outcomes |  |  |  |  |  |
| Understanding the patient's health literacy level and willingness to change unhealthy behaviors |  |  |  |  |  |

10. Institutional Emphasis (Please mark the appropriate box for each row)

| Statement | Strongly Disagree | Disagree | Neutral / Don't Know | Agree | Strongly Agree |
| --- | --- | --- | --- | --- | --- |
| Collaboration with community committees, civil affairs departments, etc. |  |  |  |  |  |
| Preventive screening and lifestyle interventions for residents |  |  |  |  |  |
| Collaboration with volunteers, schools, etc., to provide services |  |  |  |  |  |
| Disease screening and health education for patients |  |  |  |  |  |

11. Within this institution, there are established procedures for coordinating patient referrals to other departments or for consultations.
☐ Strongly Disagree ☐ Disagree ☐ Neutral / Don't Know ☐ Agree ☐ Strongly Agree

12. This institution has established procedures for referring patients to other institutions.
☐ Strongly Disagree ☐ Disagree ☐ Neutral / Don't Know ☐ Agree ☐ Strongly Agree

13. In this institution, family doctors: (Please mark the appropriate box for each row)

| Statement | Strongly Disagree | Disagree | Neutral / Don't Know | Agree | Strongly Agree |
| --- | --- | --- | --- | --- | --- |
| Obtain patients' informed consent before providing diagnosis/treatment services |  |  |  |  |  |
| Can share the same patient's treatment plan via information systems or other means |  |  |  |  |  |
| Follow the same service protocols as other doctors when providing care |  |  |  |  |  |

14. This institution has multi-disciplinary family doctor teams (e.g., including public health physicians, GPs, etc.).
☐ Strongly Disagree ☐ Disagree ☐ Neutral / Don't Know ☐ Agree ☐ Strongly Agree

15. The same patient is always served by the same contracted family doctor/team.
☐ Strongly Disagree ☐ Disagree ☐ Neutral / Don't Know ☐ Agree ☐ Strongly Agree

16. This institution's service scope is clearly defined and known by other institutions in the district.
☐ Strongly Disagree ☐ Disagree ☐ Neutral / Don't Know ☐ Agree ☐ Strongly Agree

17. Coordination status of this institution with other medical institutions within the district/county: (Please mark the appropriate box for each row)

| Statement | Strongly Disagree | Disagree | Neutral / Don't Know | Agree | Strongly Agree |
| --- | --- | --- | --- | --- | --- |
| Can reduce redundant processes (e.g., registration cards, referral appointments) through cooperation |  |  |  |  |  |
| Can coordinate the use of equipment (e.g., borrowing, leasing) |  |  |  |  |  |
| Can coordinate the flow of personnel (e.g., temporary transfers, consultations at other institutions) |  |  |  |  |  |

18. Relevant documents at the municipal/district/county level effectively promote: (Please mark the appropriate box for each row)

| Statement | Strongly Disagree | Disagree | Neutral / Don't Know | Agree | Strongly Agree |
| --- | --- | --- | --- | --- | --- |
| Collaboration between different medical institutions |  |  |  |  |  |
| Forming teams with healthcare professionals from different specialties |  |  |  |  |  |
| Contracting between patients and family doctors |  |  |  |  |  |

19. Problems often occur in sharing patient information with other medical institutions (via information systems or other means).
☐ Never ☐ Seldom ☐ Occasionally ☐ Often ☐ Always

20. Service coordination with doctors from other medical institutions is not easy to achieve.
☐ Never ☐ Seldom ☐ Occasionally ☐ Often ☐ Always

21. Problems often occur during the process of referring patients to other medical institutions (e.g., poor communication, duplicate tests/exams, discontinuous treatment).
☐ Never ☐ Seldom ☐ Occasionally ☐ Often ☐ Always

22. For patients seeking care across institutions, the following problems occur: (Please mark the appropriate box for each row)

| Statement | Never | Seldom | Occasionally | Often | Always |
| --- | --- | --- | --- | --- | --- |
| Discontinuity in medical record documentation |  |  |  |  |  |
| Duplicate medications, tests, and diagnoses |  |  |  |  |  |

23. This institution and other medical institutions within the district/county: (Please mark the appropriate box for each row)

| Statement | Never | Seldom | Occasionally | Often | Always |
| --- | --- | --- | --- | --- | --- |
| Have interoperable medical and public health information systems |  |  |  |  |  |
| Can share the same patient's lab/test results via information systems or other means |  |  |  |  |  |
| Can share patient outcomes post-discharge (e.g., satisfaction, recovery/death) via IS/other means |  |  |  |  |  |

24. You are able to obtain data on your patients' visits to other medical institutions (e.g., hospitals, other CHSCs, township hospitals).
☐ Never ☐ Seldom ☐ Occasionally ☐ Often ☐ Always

25. Your family doctor team: (Please mark the appropriate box for each row)

| Activity | Never | Seldom | Occasionally | Often | Always |
| --- | --- | --- | --- | --- | --- |
| Conducts regular follow-ups on patient needs to provide personalized services |  |  |  |  |  |
| Conducts regular follow-ups on patient satisfaction |  |  |  |  |  |
| Explains the treatment goals for each stage to the patient |  |  |  |  |  |
| Conducts regular follow-ups on patients' post-discharge status (e.g., readmission, medication adherence) |  |  |  |  |  |

26. This institution regularly checks whether family doctor teams are providing services according to treatment plans.
☐ Never ☐ Seldom ☐ Occasionally ☐ Often ☐ Always

27. Members of your family doctor team: (Please mark the appropriate box for each row)

| Statement | Never | Seldom | Occasionally | Often | Always |
| --- | --- | --- | --- | --- | --- |
| Support each other in work |  |  |  |  |  |
| Respect each other in work |  |  |  |  |  |
| Have sufficient human resources to complete daily work |  |  |  |  |  |
| Can work together effectively to complete large volumes of work in a short time |  |  |  |  |  |
| Provide help to a member when he/she is very busy |  |  |  |  |  |

28.The family doctor team uses methods like case discussions to ensure patient safety.
☐ Never ☐ Seldom ☐ Occasionally ☐ Often ☐ Always

29.Family doctor team members learn from past mistakes to ensure patient safety.
☐ Never ☐ Seldom ☐ Occasionally ☐ Often ☐ Always

30.This institution's management processes and operational systems can effectively prevent the occurrence of medical errors.
☐ Never ☐ Seldom ☐ Occasionally ☐ Often ☐ Always
